# Levosimendan and systemic vascular resistance in cardiac surgery patients: a systematic review and meta-analysis

**DOI:** 10.1038/s41598-019-56831-y

**Published:** 2019-12-30

**Authors:** Sandra Terbeck, Paul Philipp Heinisch, Armando Lenz, Jan-Oliver Friess, Dominik Guensch, Thierry Carrel, Balthasar Eberle, Gabor Erdoes

**Affiliations:** 1 Department of Anesthesiology and Pain Medicine, Inselspital, Bern University Hospital, University of Bern, Bern, Switzerland; 2 Department of Cardiovascular Surgery, Inselspital, Bern University Hospital, University of Bern, Bern, Switzerland; 3 Clinical Trials Unit, University of Bern, Bern, Switzerland

**Keywords:** Cardiac hypertrophy, Heart failure

## Abstract

Levosimendan is a potent non-adrenergic inodilator agent. The net effect of hemodynamic changes may result in a hyperdynamic state with low systemic vascular resistance. We conducted a systematic review and meta-analysis assessing hemodynamics in cardiac surgery patients treated with levosimendan. English-language literature was searched systematically from 2006 until October 2018, including randomized controlled trials and case-matched or retrospective studies providing at least two sequentially measured hemodynamic variables in adult patients who underwent cardiac surgery with cardiopulmonary bypass and were treated with levosimendan in comparison to alternative drugs or devices. Cardiac index significantly increased in the levosimendan group by 0.74 (0.24 to 1.23) [standardized mean difference (95% CI); p = 0.003] from baseline to postoperative day (POD) 1, and by 0.75 (0.25 to 1.25; p = 0.003) from baseline to POD 7, when corrected for the standardized mean difference at baseline by a multivariate mixed effects meta-analysis model. With this correction for baseline differences, other hemodynamic variables including systemic vascular resistance did not significantly differ until POD 1 [−0.17 (−0.64 to 0.30), p = 0.48] and POD 7 [−0.13 (−0.61 to 0.34), p = 0.58] between the levosimendan and the comparator group. Levosimendan increases cardiac index in patients undergoing cardiac surgery. Although levosimendan has inodilator properties, this meta-analysis finds no clinical evidence that levosimendan produces vasopressor-resistant vasoplegic syndrome.

## Introduction

**Figure 1:**
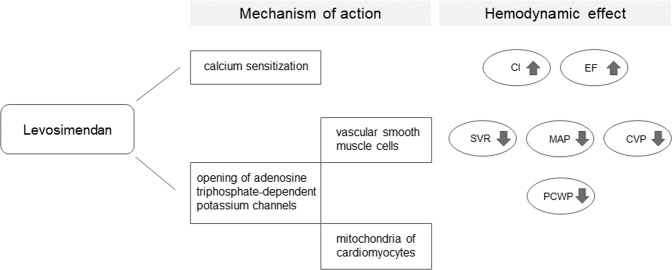
Descriptive figure illustrating mechanism of action and hemodynamic effects associated with levosimendan administration. CI, cardiac index; EF, ejection fraction; SVR, systemic vascular resistence; MAP, mean arterial pressure; CVP, central venous pressure; PCWP, pulmonary capillary wedge pressure.

Levosimendan exerts its inodilatory effects mainly through three mechanisms of action: calcium sensitization, opening of adenosine triphosphate-dependent-potassium (K_ATP_) channels in vascular smooth muscle cells and in the mitochondria of cardiomyocytes^1^. Through calcium sensitization, levosimendan has positive inotropic effects which result in an increase of cardiac output, whereas its action on K_ATP_ channels causes systemic vasodilation in both arterial and venous vascular beds (Fig. 1)^2–4^.

In recent years, levosimendan has been extensively studied in cardiac surgery. In the majority of studies, it was administered as a preoperative bolus, often immediately after anesthesia induction to exploit its preconditioning effect before surgery^5–8^. A high plasma concentration achieved in a short period of time may significantly lower systemic vascular resistance, since levosimendan acts on vascular smooth muscle cells in a dose-dependent fashion^9^.

It is known that continued preoperative intake of angiotensin-converting enzyme inhibitors or beta-blockers, low pre-operative left ventricular ejection fraction (EF), vasopressor use prior to cardiopulmonary bypass (CPB) and prolonged duration of CPB are predisposing factors for abnormally low systemic vascular resistance after cardiac surgery^10,11^. The related syndrome, post-cardiotomy vasoplegia, has been defined as hypotension in the absence of a low cardiac output state^12^, and is associated with prolonged hospital stay and increased mortality, mainly due to the resulting end-organ failure. Although the etiology of post-cardiotomy vasoplegia is probably multifactorial, and its mechanisms are not completely understood, studies ascribe a relevant contribution to the CPB circuit itself. The exposure of blood to the foreign surfaces triggers a proinflammatory response, with release of vasoactive mediators that may disturb baseline vascular reactivity and tonus. Patients with heart failure express high levels of inflammatory mediators and may therefore be particularly susceptible to vasodilatory effects of drugs^13^. It is precisely this subgroup of patients who frequently receive levosimendan in the perioperative period. Thus, short-term loading of levosimendan in the pre-CPB period, especially administered by bolus injection, may be counterproductive due to a high incidence of hypotension with subsequent need of vasopressor medication.

Recent meta-analyses on levosimendan indicate that its administration in cardiac surgery patients is associated with decreased incidence of acute renal injury and renal replacement therapy and a lower 30-d mortality, especially in those presenting with reduced left ventricular contractility^14–18^. Beyond its unequivocally proven positive inotropism, however, levosimendan’s net effect on global hemodynamics of patients undergoing cardiac surgery with CPB remains controversial.

We performed a study-level meta-analysis of the available literature. Our aim was to investigate hemodynamic parameters - especially those indicating vasoplegia - in the context of perioperative levosimendan administration in patients undergoing cardiac surgery with CPB.

A sensitivity analysis with correction of the standardized mean difference (SMD) in hemodynamic parameters at baseline was conducted to reduce heterogeneity in the baseline parameters collected by the included studies.

## Methods

**Figure 2:**
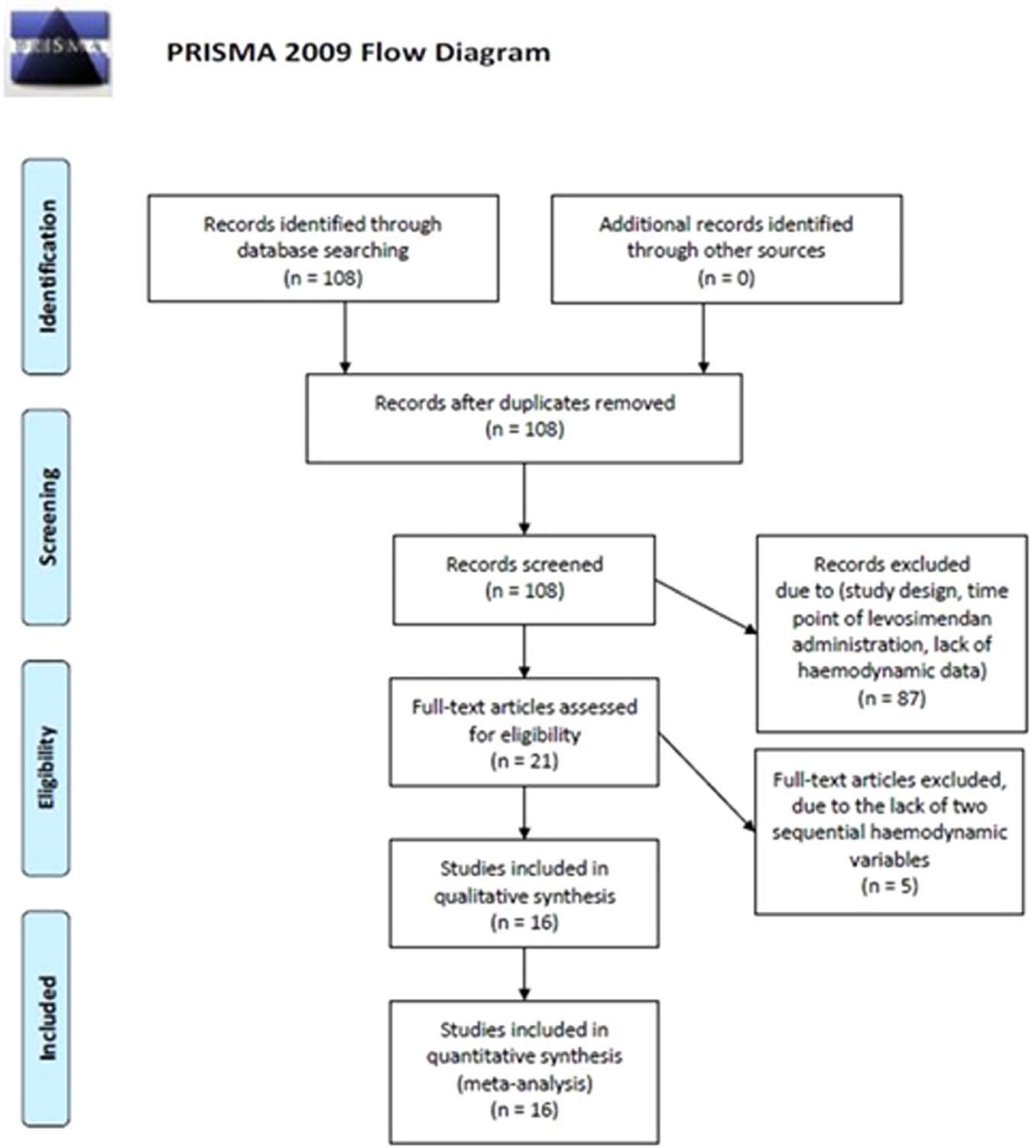
Study flow chart with numbers of abstracts screened and studies assessed for eligibility and included in the review; reasons for exclusions at each stage are also given.

This analysis included randomized controlled trials (RCTs), retrospective and case-control studies providing appropriate hemodynamic datasets from adult patients undergoing cardiac surgery with CPB, and being treated with levosimendan *vs*. a comparator. The manuscript contains all data and complies with Preferred Reporting Items for Systematic Reviews and Meta-Analyses (PRISMA) recommendations (Fig. 2, Supplements A1 and A2)^19^.

### Search strategy and eligibility criteria

Literature sources allowing unrestricted access to full texts and supplements in the US National Library of Medicine database (PUBMED), MEDLINE, EMBASE, and CENTRAL were searched systematically from 2006 until July 2018. Medical Subject Headings terms and free words referring to levosimendan in cardiac surgery were used as key words, including the following: levosimendan, cardiac surgery, cardiopulmonary bypass OR levosimendan OR milrinone OR dobutamine OR nitroglycerine OR intra-aortic balloon pump OR mechanical circulatory support AND cardiac surgery, cardiopulmonary bypass and weaning from cardiopulmonary bypass.

Key words were chosen by two authors on the basis of their own experience and other articles on similar topics. The reference lists of all selected papers were also screened for relevant studies. Eligibility criteria were: (a) English-language literature published from 2006 to October 2018, (b) study design (RCT, retrospective and case-control studies), (c) adult patient population, (d) cardiac surgery with CPB, (d) availability of at least two sequentially measured hemodynamic variables for the perioperative period, (e) levosimendan administration as a bolus and/or a continuous infusion before starting CPB, also including study subgroups with levosimendan administration as a bolus and/or a continuous infusion during CPB weaning, and (f) comparison of levosimendan to another drug or device.

### Study selection and data extraction

Two authors independently screened the titles and abstracts of the selected studies. Articles were excluded if they: (a) were not original research (e.g., case reports, editorials, reviews, congress abstracts), (b) did not provide at least two hemodynamic variables in the full-text version or in the supplement, (c) started levosimendan exclusively after cardiac surgery (after CPB weaning, post-operatively on the intensive care unit, ICU) and (d) were a duplicate. In cases where the article reported on hemodynamic variables without providing them as numbers (mean or median), the corresponding author was contacted for the original data set.

The full text versions of the articles selected by one or more of the assessors were retrieved for evaluation. Two assessors read the full texts and independently extracted the information from the selected studies. A third assessor reviewed the data extraction, and any disagreement was resolved through consensus.

### Assessment of risk of bias

The quality of the studies was evaluated with the Newcastle Ottawa Scale^20^. Two authors independently rated each study, and consensus discussion was used to resolve any dis-agreement.

### Statistical analysis

Sixteen studies were included in this meta-analysis. One study had three different levosimendan groups and another study had two different levosimendan groups. Both studies were compared only to one control group. We thus collapsed the hemodynamic endpoints of the levosimendan groups for these two studies, taking the mean of means, and the mean of the square-root of the sum of squares of standard deviations. For non-parametric results, we calculated the mean from the median, the lower quartile and the upper quartile, and the standard deviation from lower quartile, upper quartile and the number of patients, according to Wan and co-workers^21^. As our primary analysis, we calculated Hedges’ g as the standardized mean differences in order to meta-analyze endpoints among studies using the R package meta^22^. We calculated a separate meta-analysis for each endpoint and period. For sensitivity analysis we used multivariate mixed effects linear meta-analysis models, using time (baseline, a period (I) from baseline to 24 hours (inclusive) after surgery, and a period (II) from 24 hours to 7 days after surgery) as a moderator, and the individual studies as random effect, using the R package metafor^23^. We used this model to correct the standardized mean difference (SMD) at period (I), or at period (II) after surgery by the SMD at baseline. All analyses were performed using R version 3.5.0^24^. Values are numbers and percentages (n, %), or median with lower and upper quartile (m [lq, uq]).

## Results

### Description of studies

The final analysis included 16 studies (14 RCTs, 1 retrospective study and 1 case-matched study) with 1071 patients overall (Table 1). Coronary artery bypass grafting (CABG) and combined procedures (CABG and valve repair/replacement) were equally represented. The most common comparator to levosimendan was placebo (n = 13, 81%). In the majority of 19 subgroups levosimendan was administered as a pre-operative bolus of 12 mcg/kg [3, 200] and was followed by a continuous infusion of 0.1 mcg/kg/min [0.05, 0.2] (Table 2).

**Table 1:** Main descriptors of the studies included in meta-analysis.

Study	First author, year	Type	Patients	Surgery	Bolus	Infusion	Subgroups	Comparator
1.	Anastasiadis, 2016	RCT	32	Comb	No	pre-surgery	1	placebo
2.	Atalay, 2016	RCT	58	CABG	pre-surgery	pre-surgery	1	placebo
3.	Eris, 2014	RS	40	CABG	no/post-CPB	pre-surgery/post-CPB	3	placebo
4.	Sharma, 2014	RCT	40	Comb	pre-surgery	No	1	placebo
5.	Erb, 2014	RCT	33	Comb	No	pre-surgery	1	placebo
6.	Ersoy, 2013	RCT	20	Comb	pre-surgery	pre-surgery	1	placebo
7.	Lomivorotov, 2012	RCT	90	Comb	pre-surgery	pre-surgery	2	IABP
8.	Levin, 2012	RCT	252	CABG	pre-surgery	pre-surgery	1	placebo
9.	Severi, 2011	CM	22	CABG	No	pre-surgery	1	IABP
10.	Leppinkangas, 2011	RCT	24	Comb	pre-surgery	pre-surgery	1	placebo
11.	Lahtinen, 2011	RCT	200	Comb	pre-surgery	pre-surgery	1	placebo
12.	Triapepe, 2009	RCT	102	CABG	pre-surgery	No	1	placebo
13.	Eriksson, 2009	RCT	60	CABG	pre-surgery	pre-surgery	1	placebo
14.	Jarvela, 2008	RCT	24	Comb	No	pre-surgery	1	placebo
15.	Triapepe, 2006	RCT	24	CABG	pre-surgery	No	1	placebo
16.	Sahu, 2016	RCT	30	CABG	pre-surgery	pre-surgery	1	nitroglycernine

Legend: RCT, randomized controlled trial; RS, retrospective study; CM, case-matched study; CABG, coronary artery bypass grafting; Comb, combined procedure (CABG +/− valve replacement, reconstruction); CPB, cardiopulmonary bypass; IABP, intra-aortic balloon pump.

Please note that Eris *et al*. studied 3 subgroups vs. placebo (2 subgroups without a bolus but with a continuous infusion, 1 subgroup with bolus and continuous infusion post-CPB weaning) and Lomivotorov *et al*. studied 2 subgroups vs. IABP (both with bolus and continuous infusion).

**Table 2:** Summary of metadata of studies included in meta-analysis.

Metainformation	N	Median (Range)	N (%)
Year of publication	16	2012 (2006, 2016)	
Study type	16		
RCT			14 (88)
Retrospective study			1 (6)
Case-control study			1 (6)
Surgery	16		
CABG			8 (50)
Combined procedure			8 (50)
Comparator to levosimendan	16		
Placebo			13 (81)
IABP			2 (13)
nitrogycerine			1 (6)
Time point of levosimendan bolus	19*		
Pre-surgery			12 (63)
Post-CPB weaning			1 (5)
No bolus			6 (32)
Time point for levosimendan infusion	19*		
Pre-surgery			15 (79)
During CPB weaning			1 (5)**
No infusion			3 (16)

Legend: RCT, randomized controlled trial; CABG, coronary artery bypass grafting; IABP, intra-aortic balloon pump;

CPB, cardiopulmonary bypass.

* The meta-analysis includes 16 (main) studies with 19 subgroups for different levosimendan treatment strategy.

** In the study of Eris *et al*. a subgroup of 10 patients received levosimendan during CPB weaning.

Values are number, N; number and percent, N(%) or median with lower and upper quartile, median (lq, uq).

**Figure 3:**
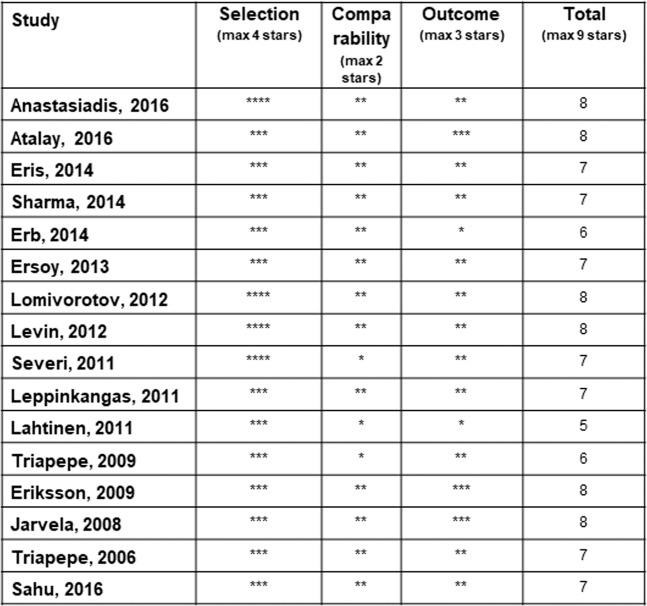
Results of the Newcastle-Ottawa Scale for evaluation of risk of bias and quality of studies included in the meta-analysis. Studies with nine stars have a low risk of bias (high quality), seven or eight stars a medium risk (moderate quality), and six or less a high risk of bias (low quality).

Risk of bias and the quality of the studies was assessed by using The Newcastle-Ottawa Scale (Fig. 3).

### Description of populations

Age and number of patients were the only baseline characteristics available in all studies. The least frequently reported characteristic was length of stay in the intensive care unit. Across all studies, baseline characteristics were similar between the levosimendan and the comparator groups (Table 3).

**Table 3:** Summary of baseline characteristics.

	Levosimendan		Comparator	
	N	Median (lq, uq)	N	Median (lq, uq)
No. of patients	16	18 (10, 127)	16	16 (10, 125)
Age (yrs)	16	65 (50, 76)	16	63 (46, 75)
Ejection fraction (%)	16	39 (18, 63)	15	43 (22, 69)
Perfusion time (min)	15	106 (73, 177)	15	122 (74, 185)
AoX time (min)	13	72 (43, 140)	13	74 (44, 137)
LOS ICU (hrs)	10	57 (25, 199)	10	53 (26, 182)
LOS Hospital (hrs)	12	271 (52, 631)	12	288 (98, 448)

Legend: AoX, aortic cross clamp; LOS, length of stay, ICU, intensive care unit.

Values are number, N and median with lower and upper quartile (lq, uq).

### Primary analysis

Reporting of endpoints differed widely among the studies and between the time points. The most frequently reported endpoints were cardiac index and pulmonary capillary wedge pressure. Mean arterial pressure was reported in nearly two thirds of the studies. Systemic vascular resistance was reported either as non-indexed (SVR) or indexed to body surface area (SVRI). Inclusion or exclusion of the studies reporting SVR (study number 6, 9, 16, Table 1) did not change the results of the sensitivity analysis. Thus, the manuscript reports the results obtained from all studies as SVR.

Central venous pressure and ejection fraction were the least frequently reported endpoints, although ejection fraction was reported in nearly all studies at baseline.

At baseline, none of the endpoints differed between the two groups. Within the first 24 hours postoperatively, levosimendan increased cardiac index, and decreased pulmonary capillary wedge pressure and SVR. This pattern prevailed until postoperative day (POD) 7, when a higher cardiac index and lower SVR were reported for patients with levosimendan than for patients treated with comparators. All other endpoints did not differ between patients receiving levosimendan and those from the comparison group (Table 4).

**Table 4:** Number of studies, standardized mean difference and p-values of random effects meta-analyses for every outcome and all three time points.

	Baseline			24 hours post surgery			7 days post surgery		
	N	SMD (95% CI)	p-value	N	SMD (95% CI)	p-value	N	SMD (95% CI)	p-value
Cardiac index	10	−0.08 (−0.30 to 0.14)	0.49	9	0.60 (0.38 to 0.82)	<0.001	8	0.61 (0.15 to 1.07)	0.009
Central venous pressure	7	0.10 (−0.16 to 0.36)	0.44	8	−0.26 (−0.53 to 0.01)	0.05	7	−0.44 (−0.89 to 0.01)	0.05
Ejection fraction	14	−0.16 (−0.40 to 0.08)	0.20	2	−0.36 (−0.94 to 0.23)	0.23	3	−0.34 (−1.18 to 0.50)	0.43
Mean arterial pressure	9	−0.00 (−0.21 to 0.20)	0.97	9	−0.10 (−0.42 to 0.23)	0.56	8	0.10 (−0.30 to 0.50)	0.63
Pulmonary capillary wedge pressure	10	−0.00 (−0.20 to 0.19)	0.96	10	−0.28 (−0.51 to −0.05)	0.02	7	−0.32 (−0.66 to 0.02)	0.06
Systemic vascular resistance/-index	9	−0.13 (−0.34 to 0.08)	0.21	8	−0.42 (−0.67 to −0.16)	0.001	7	−0.40 (−0.63 to −0.17)	<0.001

N, number, SMD standardized mean difference CI, confidence intervall.

### Sensitivity analysis

The mixed effects model meta-analysis, corrected for the standardized mean difference at baseline, confirmed most results from the primary analysis. Cardiac index increased significantly from baseline to later time periods. The other endpoints did not change significantly from baseline to the two later periods (Table 5). In sensitivity analysis, the difference of SVR between levosimendan and comparators, as described in the primary analysis section, became insignificant due to the correction for baseline differences.

**Table 5:** Standardized mean difference at 24 hours and 7 days post-surgery, corrected for the standardized mean difference at baseline, calculated by a multivariate mixed effects meta-analysis model with the time as moderator and the study as random effect.

	SMD (95% CI)	p-value	SMD (95% CI)	p-value
Cardiac index	0.74 (0.24 to 1.23)	0.003	0.75 (0.25 to 1.25)	0.003
Central venous pressure	−0.31 (−0.92 to 0.30)	0.32	−0.50 (−1.12 to 0.11)	0.11
Ejection fraction	0.23 (−0.56 to 1.02)	0.57	0.21 (−0.44 to 0.85)	0.53
Mean arterial pressure	−0.18 (−0.67 to 0.31)	0.47	0.14 (−0.36 to 0.64)	0.58
Pulmonary capillary wedge pressure	−0.32 (−0.78 to 0.14)	0.18	−0.34 (−0.81 to 0.13)	0.16
Systemic vascular resistance/-index	−0.17 (−0.64 to 0.30)	0.48	−0.13 (−0.61 to 0.34)	0.58

Legend: SMD standardized mean difference CI, confidence intervall.

**Figure 4:**
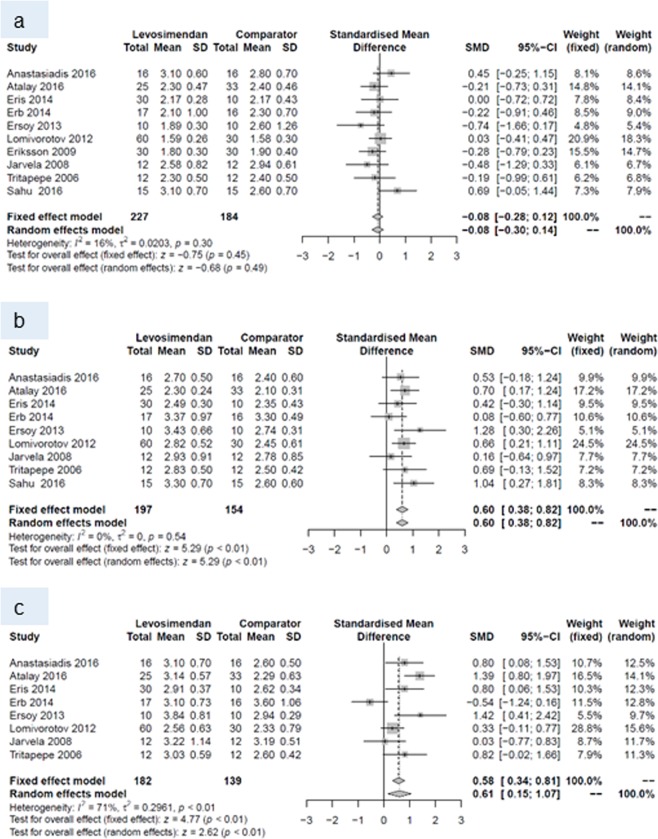
Forest plots for cardiac index: (**a**) at baseline, (**b**) during period (I) and (**c**) during period (II).

**Figure 5:**
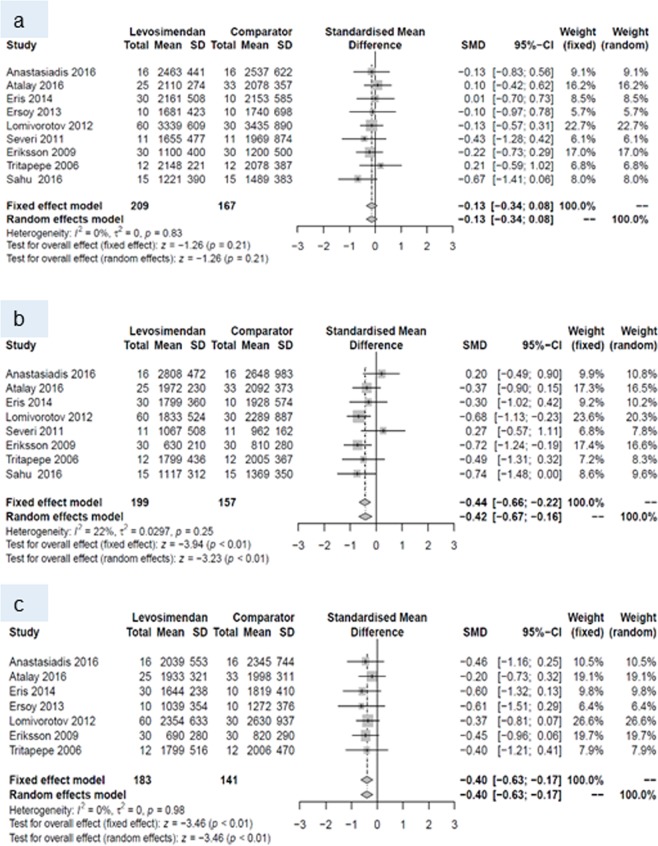
Forest plots for systemic vascular resistance: (**a**) at baseline, (**b**) during period (I) and (**c**) during period (II).

**Figure 6:**
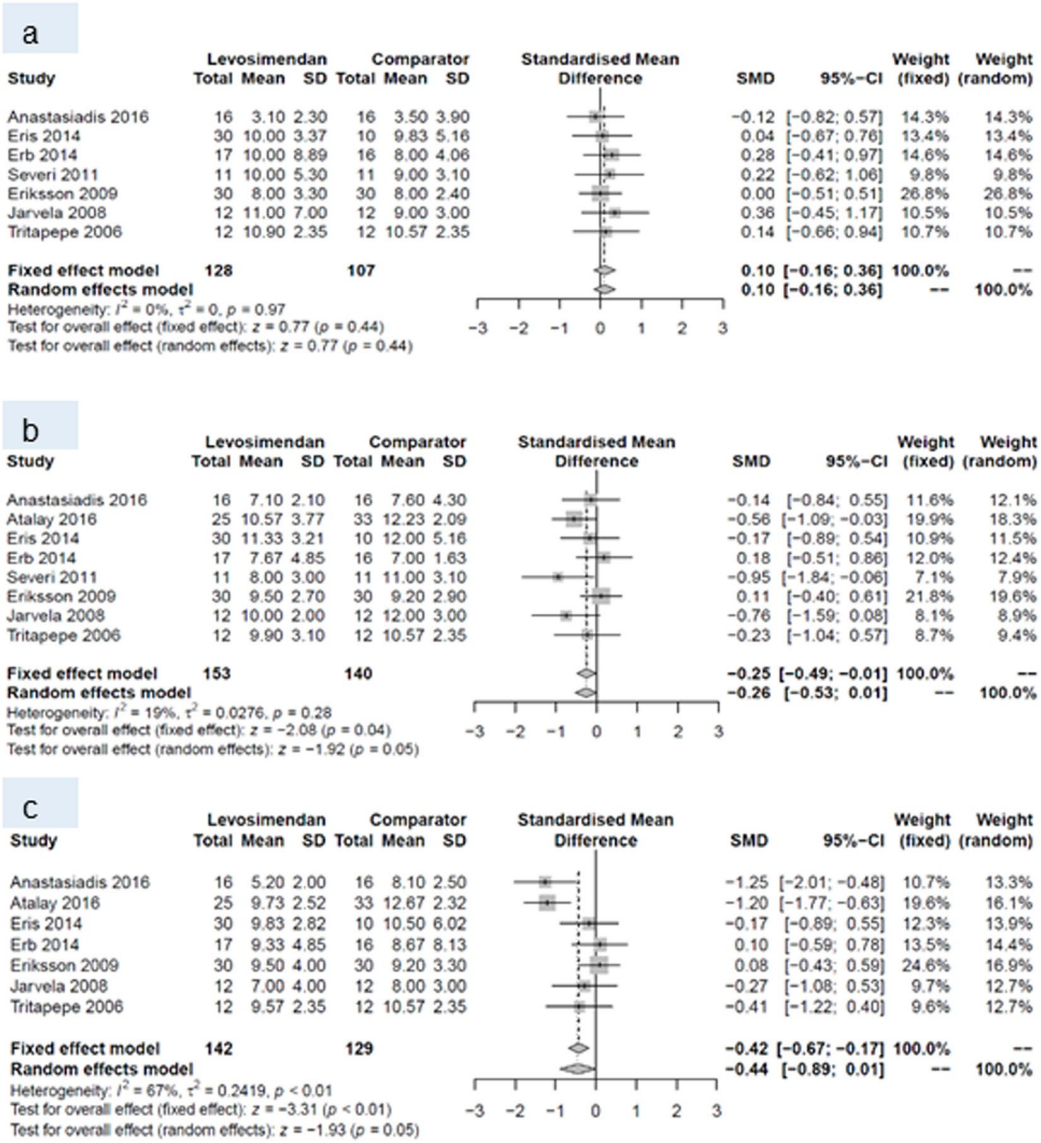
Forest plots for central venous pressure: (**a**) at baseline, (**b**) during period (I) and (**c**) during period (II).

**Figure 7:**
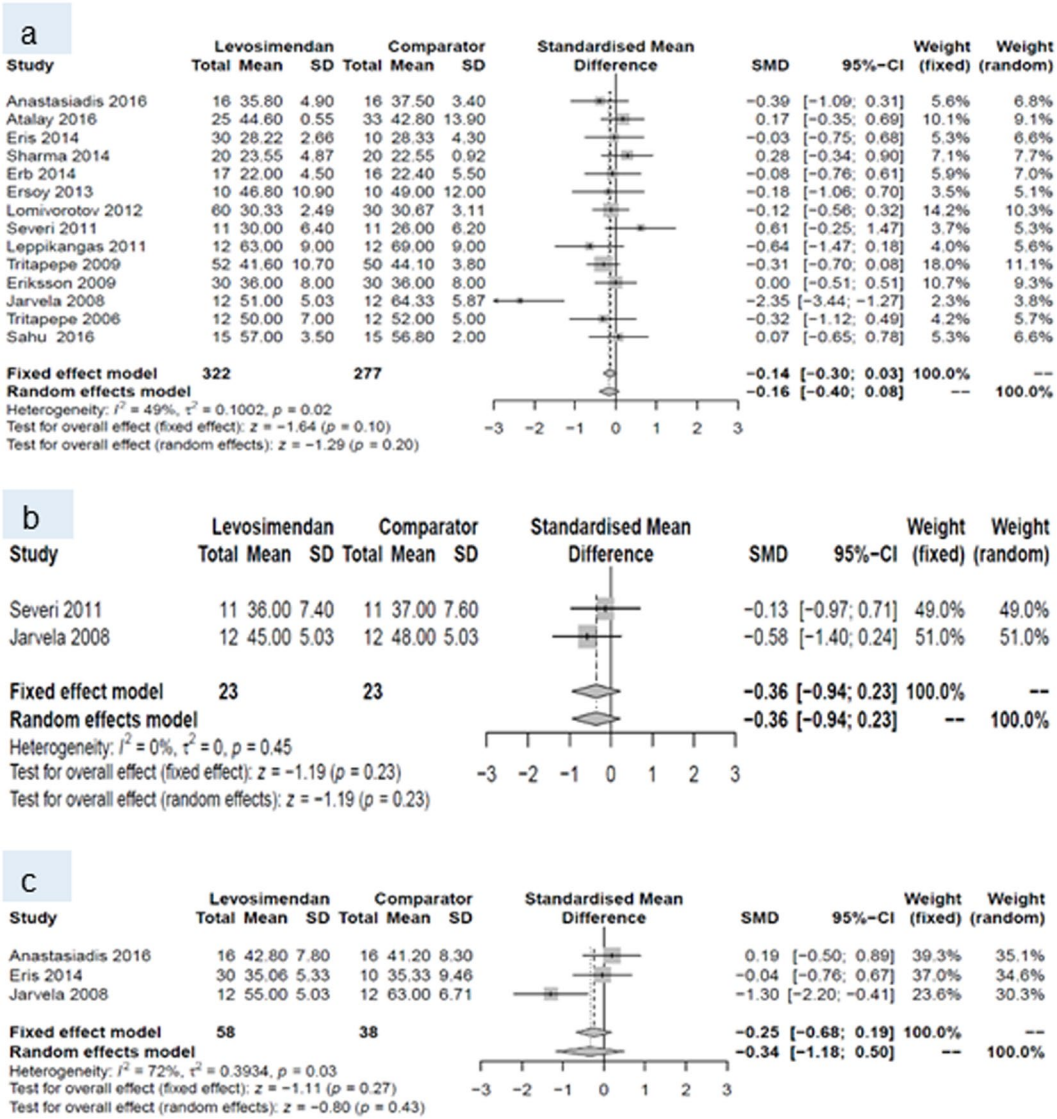
Forest plots for ejection fraction: (**a**) at baseline, (**b**) during period (I) and (**c**) during period (II).

**Figure 8:**
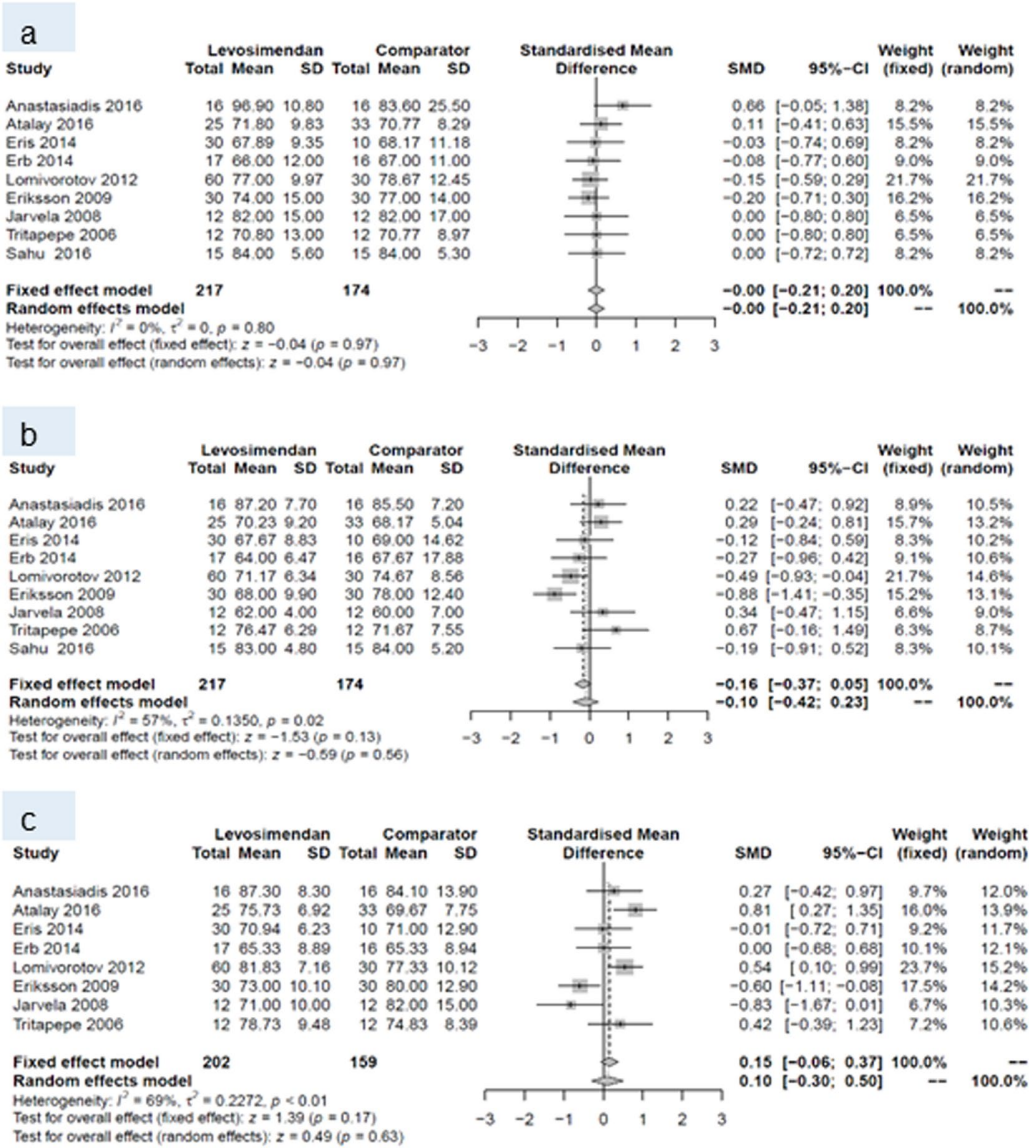
Forest plots for mean arterial pressure: (**a**) at baseline, (**b**) during period (I) and (**c**) during period (II).

**Figure 9:**
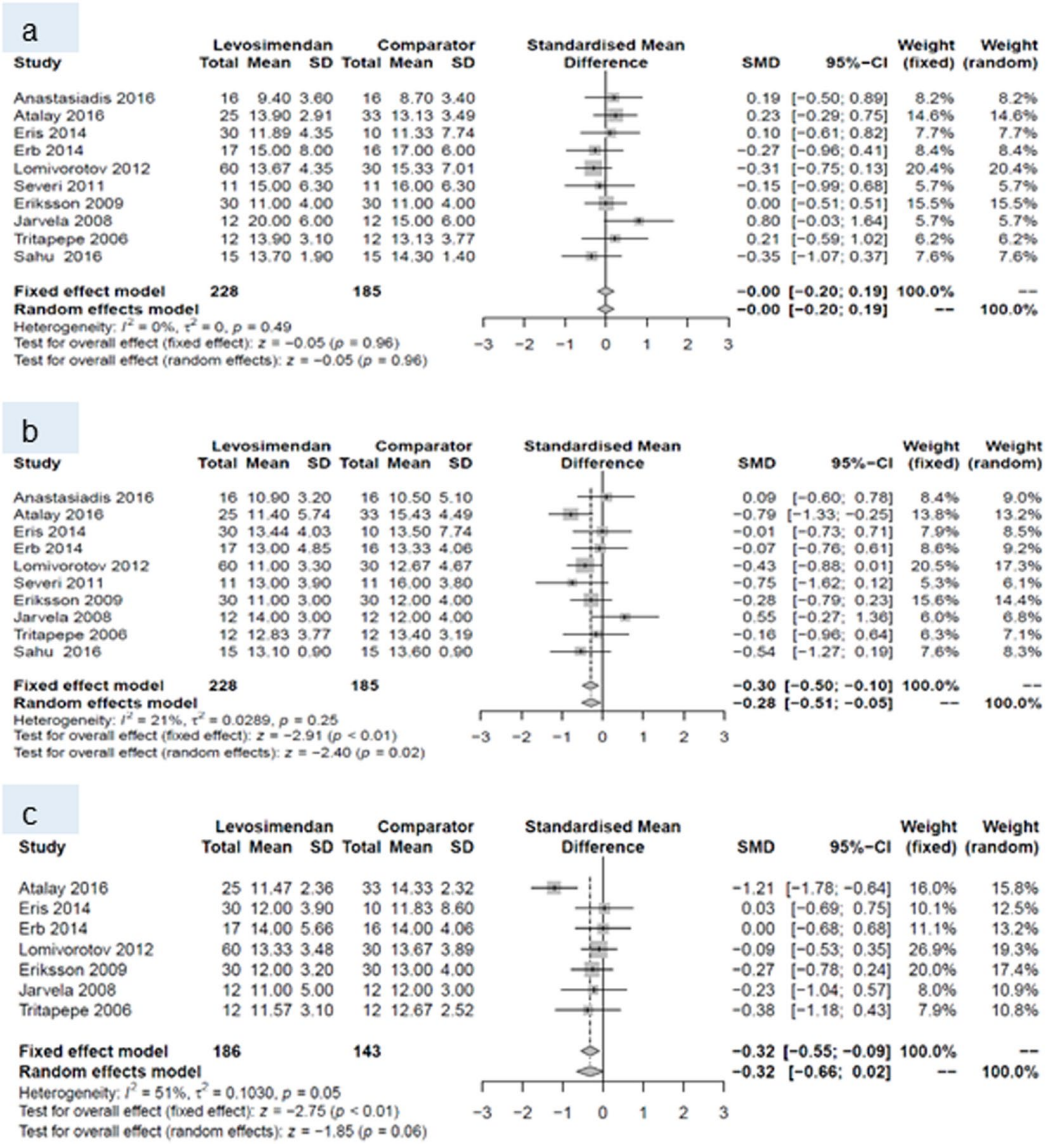
Forest plots for pulmonary capillary wedge pressure: (**a**) at baseline, (**b**) during period (I) and (**c**) during period (II).

Forest plots for cardiac index and systemic vascular resistance at baseline (a), and during periods (I) and (II) are given in Figs. 4 and 5. Forest plots for central venous pressure, ejection fraction, mean arterial pressure, pulmonary capillary wedge pressure b) during period (I) and (II) are presented in the Figs. 6, 7, 8 and 9.

## Discussion

The present systematic review and meta-analysis showed a similar pattern of hemodynamic changes across the different studies when levosimendan was prophylactically administered to adult patients undergoing cardiac surgery with CPB. During the first postoperative week, levosimendan increased cardiac index when compared to the respective comparator, whereas its use was not associated with significant reduction of systemic vascular resistance in the context of postoperative care in the studies.

During the past decade, 72 meta-analyses have been published on levosimendan in various settings, with focus on mortality, neurohormones, troponin release, renal function, length of hospital stay, pharmaco-economics etc. None of these meta-analyses focused specifically on perioperative endpoints of the hemodynamic profile. The positive inotropic action with associated increase of cardiac output, stroke volume, and ejection fraction is a well-known pharmacodynamic property of levosimendan and represents the main indication for the use of levosimendan in cardiac surgery.

Almost all studies included in this current meta-analysis showed an increase in cardiac index. The RCT by Lomivorotov showed the largest effect, with increases in cardiac index at 24 hours and 7 days after cardiac surgery^7^. This may be due to the selective inclusion of patients with low preoperative ejection fraction (<35%), and/or to the use of IABP in addition to levosimendan in a subgroup of patients. The use of IABP may have had an additional positive effect on coronary perfusion, and hence cardiac performance. Similarly, the RCTs of Anastasiadis^25^ and Atalay^26^ observed increases in cardiac index 24 hours and 7 days after cardiac surgery. Both studies, however, included only patients with mild or moderate left ventricular dysfunction undergoing CABG. In the retrospective study of Eris, the positive inotropic effect of levosimendan was still apparent at the time points of observation, however with a reduced fixed weight, mainly owing to the small subgroups (n = 10) and widely varying modalities of levosimendan administration^27^. Also, not all studies could confirm stimulatory effects of levosimendan on cardiac index with a weighting similar to other studies. Two small RCTs failed to observe a beneficial effect of levosimendan on cardiac index at their RCT’s last hemodynamic assessment point^28,29^. Reasons for this may be the small size and/or non-homogeneity of their samples, such as groups already differing in baseline EF^28^ or in the rate of post-CPB institution of mechanical circulatory support^29^. Also, in both studies levosimendan was started rather late and slowly, i.e., after anesthesia induction and without bolus, which probably delayed the onset of the drug’s inotropic action. A potential between-group difference in cardiac index may have been levelled out in the study of Järvelä by the significantly higher noradrenaline dosage, and hence afterload, in the levosimendan group^28^. In the RCT of Erb, the more frequent use of IABP and ventricular assist devices in the control group may have masked superior hemodynamic effects of levosimendan in comparison with placebo^29^.

Systemic arterial vasodilation is a genuine though often undesired effect of levosimendan, since it may be associated with relevant reductions in mean arterial pressure and total peripheral resistance^6,9,30^. Although no studies have documented increased mortality which could be causally related to levosimendan-associated hypotension^28,31^, marked arterial hypotension is undesirable during or after cardiac surgery since it may compromise end-organ perfusion^32–34^. In contrast to the primary analysis of our study, which showed a significant decrease of SVR until the seventh postoperative day, our sensitivity analysis did not confirm a significant difference in the SVR between patients treated with levosimendan and those receiving comparators. While the primary analysis focused on SVR difference between levosimendan and the comparison group at each time point, secondary analysis corrected for baseline differences. Thus, the lower SVR in the levosimendan group described by the primary analysis is due to a pre-existing baseline difference. In this context, it is conceivable that, especially in patients with preoperative IABP use, a decreased SVR was measured due to an afterload reduction. However, since our secondary analysis was corrected for baseline differences and no significant difference between levosimendan and comparator groups could be demonstrated in the further perioperative course, we assume that SVR reduction by IABP is not relevant in this setting.

Low SVR in association with the use of levosimendan in critically ill patients is frequently reported in the clinical literature, as well as in studies included in the present meta-analysis. However, there are discrepancies. Severi reported higher systemic vascular resistance in their levosimendan group when comparing it with a control group treated solely by prophylactic placement of IABP^35^. Starting study drug infusion 24 hours prior to surgery, Anastasiadis found no significant difference in SVR or MAP between their levosimendan and placebo groups until 24 hours postoperatively^25^. Similarly, the large randomized, placebo-controlled clinical trials CHEETAH^36^, LICORN^37^, and LEVO-CTS^38^ did not demonstrate an increased incidence of hypotension as an adverse event. The reasons for such discrepancies are not fully understood, but may be related to a diminished dose-dependent effect on K_ATP_ channels in vessels with low resistance when levosimendan infusion commences early in the pre-operative period, typically between 12 and 24 hours prior to CPB. Another explanation may be the rather moderate dosage of continuous levosimendan infusions (0.05–0.1 mcg/kg/min) in the respective studies or different compensatory dosages of vasopressor drugs.

The main limitation of the present study comes from missing information and inconsistencies in the reporting of hemodynamic data in the studies analyzed. Other common limitations, intrinsic in meta-analyses, are the heterogeneity of patient populations with different etiologies and stages of heart failure and comorbidities, different types of cardiac surgery (coronary artery bypass grafting vs. valve surgery) in the studies analyzed and the large variety of clinical and research protocols, which differ, for example, in terms of levosimendan dosage, administration regimens, comparators and combination therapies, with concomitant use of catecholamines, vasoactive drugs and mechanical circulatory support devices.

In conclusion, this meta-analysis and systematic review confirms that perioperative treatment with levosimendan improves cardiac index in adult patients undergoing cardiac surgery with CPB. As a new aspect, however, this current review finds no clinical evidence that levosimendan produces vasopressor-resistant vasoplegic syndrome. Thus, objections to the use of levosimendan in cardiac surgery appear unjustified if they are based solely on the fear of triggering postcardiotomy vasoplegic syndrome.

## Supplementary information

Supplement A1.

Supplement A2.
