# Supplementary material for: Levosimendan and systemic vascular resistance in cardiac surgery patients: a systematic review and meta-analysis
Source: Sci Rep. 2019 Dec 30;9:20343. doi: 10.1038/s41598-019-56831-y (PMC6937247; doi:10.1038/s41598-019-56831-y)
Supplement: Supplementary file 2 — Supplement A2. [file 41598_2019_56831_MOESM2_ESM.pdf]

Levosimendan and systemic vascular resistance in cardiac surgery patients: a systematic review and meta-analysis

Sandra Terbeck, MD, Paul Philipp Heinisch, MD, Armando Lenz, PhD, Jan-Oliver Friess, MD, Dominik Guensch, MD, Thierry Carrel, MD, PhD, Balthasar Eberle, MD, and Gabor Erdoes, MD, PhD

Supplement A2: Prisma-DTA Abstract checklist

Section/topic	#	PRISMA-DTA for Abstracts Checklist item	Reported on page #
**TITLE and PURPOSE**			
Title	1	Identify the report as a systematic review (+/- meta-analysis) of diagnostic test accuracy (DTA) studies.	**2**
Objectives	2	Indicate the research question, including components such as participants, index test, and target conditions.	**2**
**METHODS**			
Eligibility criteria	3	Include study characteristics used as criteria for eligibility.	**2**
Information sources	4	List the key databases searched and the search dates.	**2**
Risk of bias & applicability	5	Indicate the methods of assessing risk of bias and applicability.	**2**
Synthesis of results	A1	Indicate the methods for the data synthesis.	**2**
**RESULTS**			
Included studies	6	Indicate the number and type of included studies and the participants and relevant characteristics of the studies (including the reference standard).	**2**
Synthesis of results	7	Include the results for the analysis of diagnostic accuracy, preferably indicating the number of studies and participants. Describe test accuracy including variability; if meta-analysis was done, include summary results and confidence intervals.	**2**
**DISCUSSION**			
Strengths and limitations	9	Provide a brief summary of the strengths and limitations of the evidence	**2**
Interpretation	10	Provide a general interpretation of the results and the important implications.	**2**
**OTHER**			
Funding	11	Indicate the primary source of funding for the review.	**2**
Registration	12	Provide the registration number and the registry name	**2**

Adapted From: McInnes MDF, Moher D, Thombs BD, McGrath TA, Bossuyt PM, The PRISMA-DTA Group (2018). Preferred Reporting Items for a Systematic Review and Meta-analysis of Diagnostic Test Accuracy Studies: The PRISMA-DTA Statement. JAMA. 2018 Jan 23;319(4):388-396. doi: 10.1001/jama.2017.19163.
